# Philadelphia Almshouse Infirmary

**Published:** 1831-01

**Authors:** 


### PHILADELPHIA ALMSHOUSE INFIRMARY.

### *Cases of Mania a Potu.* By Jesse Carter, M.D.*

Robert Peacock, æt. thirty-five, had mania a potu once, has been drinking very hard for the last month, and slept out nearly every night. Admitted May 6th. Symptoms on admission were those of acute pleuritis, connected with epileptic convulsions (for the first time in his life,) which were relieved by cupping, blistering, purging, shaving the head, and constant application of cold water, with the occasional use of the following: ℞. Lac. Assafœtida, ℥vj.; Acet. Tinct. Opii† ʒij. But sleep not succeeding, on the third day mania a potu became developed.

9th.—Is very wild, constantly imagines that there is a rattlesnake running across the room, and persons with guns endeavouring to shoot it; occasionally jumping up and getting into the corner, in order, he says, to escape the shot which are fired at the snake.

* American Journal of Med. Sciences.

† Next to opium, Dr. Carter has found assafœtida the most valuable remedy. It is stated by high authority, that, if we procure sleep in this disease, all danger is at an end; but Dr. C.'s observations lead him to a different conclusion: he has often seen patients awake from a long and continued sleep, as delirious as ever, and the disease, notwithstanding, proceed to a fatal termination. The disease is more violent and intense in hot weather. "In the Philadelphia Almshouse, between the 1st of November, 1828, and the 1st of February following, out of seventy cases, there were only nine deaths, being a little more than one in eight; whereas, between June 10th, 1829, and September 10th following, out of seventy-five cases, there were eighteen fatal, being about the proportion of one to four."

Head feels heavy, skin rather dry, pupils contracted, complains of pain about the umbilicus; pulse is full, with some force, eighty per minute. Ordered cups to seat of pain, and to the temples; four grains of opium every two hours.

Afternoon.—Is still very wild, face greatly flushed, covered with profuse perspiration; has only taken four grains of opium. Ordered cups to head to be repeated, and opium continued.

10th.—Took only four grains; went to sleep very shortly after the cups were applied, and slept nearly all night, and on waking was more quiet and rational; bowels not open for two days past. Ordered ℞. Calomel, grs. x.; Opium, gr. j.; and large bottle of porter—ten gtts. of acet. opii in each glass.

Evening.—Bowels not open, complains of slight pain in the head, other symptoms the same. Ordered cups again to head; injection of senna and salts; and xx. gtts. acet. opii every hour.

11th.—Took one hundred gtts. of Acet. Opii, slept about four hours, bowels opened three times. Complains of nausea, and pain on pressure in the epigastric region. Ordered cups to it, and the following:—Calomel, grs. iv.; Opium. grs. ij.; q. b. h.

Evening.—Has taken twelve grains of calomel, bowels once opened, head is excited, pulse has rather more force. Omit powders, and apply cups to head.

12th.—Slept some last night, head is still a little flighty; says he saw twenty persons last night, and is now occasionally talking to persons whom he imagines are present, though when spoken to he answers very correctly; other symptoms are diminished. Ordered cups to head.

13th.—Slept some towards morning, still flighty, head hot, with some uneasiness. Ordered cups to be repeated to head.

14th.—Is now asleep, and slept nearly all last night.

15th.—Slept today and yesterday; says that he never felt better. Was soon afterwards discharged eured.

Case II.—Margaret Robards, æt. forty, has been long exposed to bodily and mental suffering. In order to drown them, she has been accustomed to drink excessively. For several weeks past she has been travelling, and is now very much exhausted. Has not slept any for several nights. Was admitted May 26th. She is now quite wild, but appears disposed to sleep. Ordered xl. gtts. Acet. Opii.

27th. Has slept none, was very wild all night; pulse is small, and rather feeble, ninety-six per minute, pupils contracted, skin moist, bowels open. Ordered cold to head; Assafœtida Mixt. q. b. h.; and blisters to the arms.

Evening.—Is very restless, afraid that we are going to injure her; epigastrium very tender upon pressure; tongue inclined to dryness; other symptoms the same. Ordered mustard plaster to stomach, and xxx. gtts. Acet. Opii. q. b. h.

28th.—Was soon relieved by the plaster, and after taking sixty gtts. of Acet. Opii. went to sleep, and continue so now.

Afternoon.—Slept about eight hours: is still wild and restless, pulse small and weak. Ordered Acet. Opii, thirty gtts. q. b. h., to be alternated with the ammoniated julep, and with the free use of whiskey.

Evening.—No alteration; has taken sixty gtts. Acet. Opii. Ordered black drop to be omitted, and to take Pulv. Opii q. b. h., with continuance of treatment.

Ten P.M.—Head considerably excited by the opium and stimuli, face flushed, scalp hot, skin cold and dry. Omit opium; rub the skin with decoct. cantharides and turpentine; apply cold to head, and blisters to ankles.

29th.—Slept very little last night, but is now asleep.

30th.—Slept very well last night, is now composed and rational. Is directed, in addition, some peperpot.

From this time she gradually recovered her strength by the continuance of the generous diet, and occasional use of the diffusible stimulants, and was discharged cured in the course of a fortnight.

Case III.—George Dormont, æt. thirty-five, admitted May 16th, 1829. Very intemperate; has been drinking very hard for two weeks, and has not slept any for the last three nights. Is now quite wild, imagines there are persons in the room who wished to shoot him; skin warm and rather dry; pulse full and soft, eighty per minute; head hot; tongue moist and clean; pupils slightly dilated; bowels not open; considerable tenderness in the epigastrium. Ordered four cups to head, and four to epigastrium, with cold applications to head, injection of senna and salts, immediately after, four grains of opium, and continue three every two hours.

16th.—Has taken sixteen grains of opium; cups bled very freely; slept about two hours this morning; is still extremely wild; pupils contracted; bowels not open. Ordered, repeat cups to head, and senna injection.

Afternoon.—Resume opium, four grains every two hours.

Evening.—Having considerable cerebral tendency, six cups were repeated to head, and opium continued.

17th.—Took yesterday twenty-eight grains of opium; is more quiet; bowels only scantily opened. Ordered senna tea.

Evening.—Pulse soft and frequent; head free from cerebral excitement; skin moist and warm. The opium was resumed.

18th.—Took eighteen grains of opium; slept very little; is very wild; says his head feels very well; pulse full, and of a considerable force. Ordered, venesection ℥x. and continue opium.

Afternoon.—The blood drawn is cupped and buffed, with very little serum; pulse has risen in force. The orifice was again opened, and blood permitted to run until its tension was reduced, which required about ℥viij.; continue opium.

19th.—Slept at intervals three hours last night; took yesterday thirty-two grains of opium; pulse is frequent and soft, 108 per minute; is much calmer. Ordered a little porter.

Evening.—Has slept six hours during the day; is very restless; pulse has become rather tense. Ordered venesection ℥viij. and resume opium.

20th.—Took yesterday sixteen grains of opium; more quiet, and slept about four hours during the night; some excitement still remaining. Ordered senna tea.

24th.—For several days he has been purged, and taken from forty to sixty drops of Acet. Opii at bedtime; has slept generally very well; still some cerebral irritation; pulse full, with considerable tension, seventy-two per minute; mind yet wandering; is very fearful of dying. Ordered cups freely to head, which not relieving the excitement, he was in the evening bled ℥xij. which reduced the volume, and increased its frequency to ninety.

25th.—Mania still perceptible; head feels heavy and dull; tongue is slightly furred and red at the edges. Ordered ℞. Blue mass, gr. v. at night.

27th.—Became quite wild. Is now directed a showerbath, which, though repeated, was only of temporary benefit, and as the pulse still possessed some force and tension, he was bled ℥xiv.; omit the opiate and continue blue mass.

From this time, by the occasional use of cups, cathartics, and perpetual blisters to the back of the neck, and the steady continuance of the blue pill, his symptoms became gradually alleviated, when in about ten days gentle ptyalism supervened, which entirely dissipated his mania, and he was in a very short time restored to his usual health.

